# Optimizing home visits through machine learning for preventing peritoneal dialysis-associated peritonitis: a proof of concept study and results from PDOPPS

**DOI:** 10.1093/ckj/sfae136

**Published:** 2024-04-30

**Authors:** Tossaporn Sapsitthikul, Krit Pongpirul, Talerngsak Kanjanabuch, Piyatida Chuengsaman, Proadpran Punyabukkana, Ploy N Pratanwanich, Panus Sawetpiyakul, Dhammika Leshan Wannigama, Paweena Susantitaphong, Natavudh Townamchai, Yingyos Avihingsanon, Jeffrey Perl, David W Johnson, Roberto Pecoits-Filho, Somchai Eiam-Ong, Kriang Tungsanga, Jiruth Sriratanaban, Thailand PDOPPS Steering Committee, Kearkiat Praditpornsilpa, Kriang Tungsanga, Piyaporn Towannang, Kanittha Triamamornwooth, Pichet Lorvinitnun, Sirinart Raweewan, Jitta matawon, Nisa Thongbor, Suchai Sritippayawan, Nipa Aiyasanon, Guttiga Halue, Donkum Kaewboonsert, Pensri Uttayotha, Kittisak Tangjittrong, Wichai Sopassathit, Salakjit Pitakmongkol, Ussanee Poonvivatchaikarn, Bunpring Jaroenpattrawut, Somphon Buranaosot, Sukit Nilvarangkul, Warakoan Satitkan, Wanida Somboonsilp, Pimpong Wongtrakul, Ampai Tongpliw, Anocha Pullboon, Chanchana Boonyakrai, Montha Jankramol, Surapong Narenpitak, Piyarat Rojsanga, Apinya Wechpradit, Sajja Tatiyanupanwong, Chadarat Kleebchaiyaphum, Wadsamon Saikong, Worauma Panya, Siriwan Thaweekote, Sriphrae Uppamai, Jarubut Phisutrattanaporn, Sirirat Sirinual, Natchaporn Doenphai, Setthapon Panyatong, Puntapong Taruangsri, Boontita Prasertkul, Thanchanok Buanet, Rutchanee Chieochanthanakij, Panthira Passorn, Niwat Lounseng, Rujira Luksanaprom, Angsuwarin Wongpiang, Metinee Chaiwut, Worapot Treamtrakanpon, Ruchdaporn Phaichan, Peerapach Rattanasoonton, Wanlaya Thongsiw, Narumon Lukrat, Sayumporn Thaitrong, Phichit Songviriyavithaya, Yupha Laoong, Niparat Pikul, Uraiwan Parinyasiri, Korawee Sukmee, Tossaporn Sapsitthikul, Hathairat kosing, Hataishanok Boonprasert, Napassaporn Semtapra, Tharaporn Sudjai, Piyarat kaewprasert, jarika homsang, Sudawan Iamngamsup, Pornpan Auamim, Nutchaprang Bumrungwat, Piyatida Chuengsaman, Kandasud Sriudom, Sumalee Chankalee, Banphaeo-Charoenkrung

**Affiliations:** Division of Nephrology, Department of Medicine, Faculty of Medicine, Chulalongkorn University and King Chulalongkorn Memorial Hospital, Bangkok, Thailand; Center of Excellence in Preventive and Integrative Medicine and Department of Preventive and Social Medicine, Faculty of Medicine, Chulalongkorn University, Bangkok, Thailand; Department of International Health, Johns Hopkins Bloomberg School of Public Health, Baltimore, MD, USA; Department of Infection Biology & Microbiomes, Faculty of Health and Life Sciences, University of Liverpool, Liverpool, UK; Division of Nephrology, Department of Medicine, Faculty of Medicine, Chulalongkorn University and King Chulalongkorn Memorial Hospital, Bangkok, Thailand; Center of Excellence in Kidney Metabolic Disorders, Faculty of Medicine, Chulalongkorn University, Bangkok, Thailand; Peritoneal Dialysis Excellent Center, King Chulalongkorn Memorial Hospital, Bangkok, Thailand; Banphaeo-Charoenkrung Peritoneal Dialysis Center, Banphaeo Dialysis Group, Banphaeo Hospital, Bangkok, Thailand; Department of Computer Engineering, Faculty of Engineering, Chulalongkorn University, Bangkok, Thailand; Department of Mathematics and Computer Science, Faculty of Science, Chulalongkorn University, Bangkok, Thailand; Chula Intelligent and Complex Systems Research Unit, Chulalongkorn University, Bangkok, Thailand; International School of Engineering, Faculty of Engineering, Chulalongkorn University, Bangkok, Thailand; Department of Infectious Diseases and Infection Control, Yamagata Prefectural Central Hospital, Yamagata, Japan; Department of Microbiology, Faculty of Medicine, Chulalongkorn University, King Chulalongkorn Memorial Hospital, Thai Red Cross Society, Bangkok, Thailand; Center of Excellence in Antimicrobial Resistance and Stewardship, Faculty of Medicine, Chulalongkorn University, Bangkok, Thailand; School of Medicine, Faculty of Health and Medical Sciences, The University of Western Australia, Nedlands, Western Australia, Australia; Biofilms and Antimicrobial Resistance Consortium of ODA Receiving Countries, The University of Sheffield, Sheffield, UK; Pathogen Hunter's Research Collaborative Team, Department of Infectious Diseases and Infection Control, Yamagata Prefectural Central Hospital, Yamagata, Japan; Division of Nephrology, Department of Medicine, Faculty of Medicine, Chulalongkorn University and King Chulalongkorn Memorial Hospital, Bangkok, Thailand; Center of Excellence for Metabolic Bone Disease in CKD Patients, Faculty of Medicine, Chulalongkorn University, Bangkok, Thailand; Division of Nephrology, Department of Medicine, Faculty of Medicine, Chulalongkorn University and King Chulalongkorn Memorial Hospital, Bangkok, Thailand; Division of Nephrology, Department of Medicine, Faculty of Medicine, Chulalongkorn University and King Chulalongkorn Memorial Hospital, Bangkok, Thailand; St. Michael's Hospital, Toronto, ON, Canada; Department of Kidney and Transplant Services, Princess Alexandra Hospital, Woolloongabba, Brisbane, Queensland, Australia; Australasian Kidney Trials Network, University of Queensland, Brisbane, Australia; Translational Research Institute, Brisbane, Australia; Arbor Research Collaborative for Health, Ann Arbor, MI, USA; Division of Nephrology, Department of Medicine, Faculty of Medicine, Chulalongkorn University and King Chulalongkorn Memorial Hospital, Bangkok, Thailand; Division of Nephrology, Department of Medicine, Faculty of Medicine, Chulalongkorn University and King Chulalongkorn Memorial Hospital, Bangkok, Thailand; Center of Excellence in Preventive and Integrative Medicine and Department of Preventive and Social Medicine, Faculty of Medicine, Chulalongkorn University, Bangkok, Thailand

To the Editor,

Peritonitis, a frequent complication in peritoneal dialysis (PD), concerns both patients and nephrology teams due to its impact on patient and technique survivals, as well as quality of life [1]. Investigating artificial intelligence measures in the field of PD is essential [2], potentially aiding in organizing preventive measures such as home visits, especially in centers where the policy is to visit the patient's home [3]. The objective of this proof of concept study is to demonstrate that a machine-learning (ML) algorithm assists in scheduling home visits based on the risk of peritonitis.

Fifteen clinical and laboratory variables from the Thai Peritoneal Dialysis Outcome and Practice Patterns Study (PDOPPS), an international prospective cohort study aimed at identifying optimal practices for maintenance PD, were used to train the ML algorithm for generating a risk-based patient visit sequence. This dataset comprised 546 patients, 50% male, with a mean age of 56 ± 14 years, a peritonitis rate of 21%, and a mean risk score of 0.29 ± 0.18 across 22 sites. The study rationale and methods of Thailand PDOPPS have been previously published [4]. The top five features of importance in the algorithm included hemoglobin standard deviation (SD), bicarbonate trend, potassium SD, sodium last, and diastolic blood pressure trend. Subsequently, the ML-generated risk-based sequence was validated using data from two hospitals: (i) Banphaeo Hospital (Hospital B) with 753 patients, 53% male, mean age 59 ± 13 years, peritonitis rate 32%, and mean risk score 0.23 ± 0.16, and (ii) Samutprakan Hospital (Hospital S) with 238 patients, 50% male, mean age 56 ± 14 years, peritonitis rate 18%, and mean risk score 0.19 ± 0.11.

The ML algorithm generated risk scores for estimating the likelihood of peritonitis in patients undergoing PD. These risk scores were derived from regression models, with high-risk patients having scores near 1.00 and low-risk patients having scores near 0.00. The performance of ML algorithm was evaluated using receiver operating characteristic (ROC) and precision-recall (PR) curves, resulting in an ROC of 0.74 and a PR of 0.42. An ROC of 0.74 suggests moderate discrimination ability of the ML algorithm in distinguishing between high-risk and low-risk patients. Similarly, a PR of 0.42 indicates a moderate precision in identifying high-risk cases among those predicted by the model. Additionally, the comparison between risk-based sequencing and traditional home-visit methods in Hospital B and Hospital S settings revealed significant differences. The correlation coefficients (Spearman's *r*) for Hospital B and Hospital S were −0.02 and 0.16, respectively. A correlation coefficient close to zero indicates little to no correlation between the two methods, suggesting that their approaches to patient prioritization diverge significantly in both settings. For example, patients at Hospitals B and S with high-risk scores (0.76 and 0.60) were advanced from 18th and 17th in the traditional sequences to sixth and first, respectively, in the priority list. Conversely, patients at Hospitals B and S with low-risk scores (0.14 and 0.60) were moved down from first and second in the traditional sequences to 461st and 54th, respectively, in the risk-based sequence (Fig. 1).

**Figure 1:**
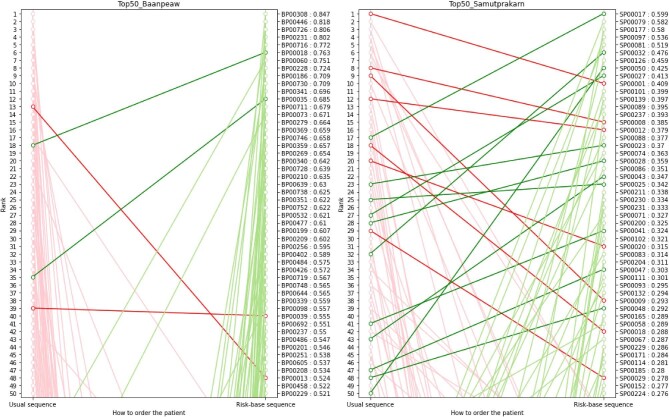
Top 50 sequences prioritized for visiting PD patients of Hospital B and Hospital S.

Although this ML-generated risk-based patient visit tool is innovative, some limitations are worth mentioning. First, several variables known to be associated with an increased risk of peritonitis (e.g. comorbidities, residual kidney function, PD vintage) were omitted due to missing data since the collection of PD patient data was conducted during the COVID-19 pandemic, potentially affecting the model's accuracy [5]. Second, the traditional home visits conducted by Hospitals B and S might not have been decided at random or by the calendar time; the sequence might have been based on the nurses' subjective assessments of the patients’ needs. Third, based on the limited evidence available to date, the effect of home visits on reducing peritonitis risk remains uncertain. The significant differences observed between risk-based sequencing and traditional home-visit methods underscore the potential benefits of adopting data-driven approaches to patient care management. However, further research may be needed to enhance the predictive accuracy and practical utility of the ML algorithm in clinical settings to reduce peritonitis rate.

In conclusion, our ML model, derived from a robust dataset, demonstrates promise in prioritizing PD patient home visits. The algorithm should be considered as a tool among other tools that help nephrology teams identify patients at risk of peritonitis. Further studies are needed to explore its effectiveness in enhancing PD care outcomes.

## Supplementary Material

sfae136_Supplemental_File

## Data Availability

The data that support the findings of this study are available from the corresponding author upon reasonable request.
